# The impact of school bullying on the mental health of boarding secondary school students: the mediating roles of school belongingness and resilience

**DOI:** 10.1186/s13034-025-00887-4

**Published:** 2025-03-26

**Authors:** Xiaoyang Liu, Ling Zhang, Yijin Wu, You Xin, Ye Wang, Xiaoyou Su

**Affiliations:** School of Population Medicine and Public Health, Chinese Academy of Medical Sciences & Peking Union Medical College, 31 BeiJiGe San Tiao, Dongcheng District, Beijing, China

**Keywords:** School bullying, Mental health, Boarding students, School belongingness, Resilience, Mediation effects

## Abstract

**Background:**

School bullying has become a significant educational and public health issue worldwide. Boarding secondary school students, who live within the school environment and away from familial support, are particularly vulnerable. This study aims to address this gap by examining the impact of school bullying on the mental health of boarding secondary school students and exploring the mediating roles of school belongingness and psychological resilience.

**Methods:**

A stratified random cluster sampling method was used to survey students from 4 boarding secondary schools in Hebei Province, China. A total of 1,560 valid responses were obtained from 1,700 questionnaires distributed. Participants provided self-reported data based on the structured questionnaire. Descriptive statistics summarized the data, and Chi-square tests, t-tests, and ANOVA were used to examine demographic differences. Pearson correlation analysis assessed relationships among bullying, mental health, school belongingness, and resilience. Path analysis was performed to test the hypothesized mediation model with 5000 bootstrap sampling. All statistical analyses were conducted using SPSS 26.0 and Amos 28.0.

**Results:**

Abnormal mental health status was reported by 33.8% of students, with 23.3% exhibiting mild abnormalities, 8.7% moderate abnormalities, 1.3% severe abnormalities, and 0.1% exhibiting very severe abnormalities. Verbal bullying, physical bullying, and social bullying were reported by 26.9%, 10.1%, and 15.4% of students, respectively, with higher prevalence among males (*P* < 0.05). Students who experienced bullying showed significantly higher rates of mental health problems (*P* < 0.001). Mediation analysis indicated that school belongingness [β = 0.017 (0.014–0.020)] and psychological resilience [β = 0.002 (0.001–0.003)] partially mediated the effect of bullying on mental health, accounting for 35.7% and 3.2% of the total effect, respectively.

**Conclusion:**

This study reveals the significant impact of school bullying on mental health among boarding secondary school students in Hebei Province, China. The findings underscore the critical roles of school belongingness and psychological resilience as mediators in the relationship between bullying and mental health outcomes. The study highlights the need for comprehensive intervention strategies that promote a positive school climate, strengthen student-teacher relationships and enhance peer support systems to foster a strong sense of school belongingness and resilience, ultimately improving overall student well-being.

## Introduction

As a complex public health issue, bullying was first defined by Dan Olweus as a deliberate, repeated or long-term exposure to negative acts performed by a person or group of persons regarded of higher status or greater strength than the victim [1, 2]. Bullying can manifest verbally like threat or insult, physically like assault, relationally like gossip or isolate others, or electronically like cyberbullying. Globally, 30-37.4% of adolescents aged 12–18 report being bullied during school [3, 4], and there is substantial regional variation in the prevalence of bullying experienced [5]. A 2019 UNESCO report found bullying prevalence ranged from 30.3 to 48.2%, with the highest rates in sub-Saharan Africa (48.2%) and North Africa (42.7%) [6]. In mainland China, national studies have shown prevalence of self-reported bullying victimization ranged from 26.1–52.1% [7, 8].

The impact of bullying on adolescents is profound and multifaceted. Evidence suggests that victims of frequently bullying may experience physical symptoms such as colds, headaches, stomachaches, and sleep disturbances, as well as internalizing problems like anxiety, depression, and harmful behaviors like substance use, suicide attempts, and self-harm [9, 10]. Perpetrators of bullying also face increased risks of psychosocial maladjustment, such as substance use, depression, criminal behavior, and antisocial tendencies [11]. Similarly, the impact of bullying is observed among boarding school students.

Boarding schools, which originated from British public schools and has practiced nearly 40 years in China, integrate students’ personal and academic lives through a closed management model [12]. While some argued this model promotes academic achievement, self-discipline, and peer relationships, others suggested it may harm students’ physical and mental well-being due to reduced family influence and increased pressure [12, 13]. A cross-sectional study found that the prevalence of depression, anxiety and stress among secondary boarding school students were 39.7%, 67.1% and 44.9%, respectively [14]. Furthermore, boarding students may experience higher rates of bullying due to extended peer interactions in a closed environment, coupled with separation from parents, former friends, and familiar surroundings [15, 16]. A strong sense of school belongingness may be a key factor in mitigating these negative effects.

School belongingness refers to students’ beliefs and feeling that they are personally accepted, respected, included, and supported by others in the school social environment [17]. It encompasses the quality of students’ connections with their teachers and peers and the extent to which they perceive the school as a protective environment. A strong sense of school belongingness fostered positive emotions, increasing satisfaction and overall happiness [18]. Research found that school belongingness is associated with students’ academic performance and psychological adjustment, mediating the impact of academic stress and depression [19], and buffering against the negative effects of bullying [20]. Higher levels of school belongingness were associated with fewer depressive symptoms [21] and lower social anxiety. For boarding students, a strong sense of school belongingness mediated reduced depression and anxiety, despite experiencing higher rates of bullying [15]. In addition to school belongingness, resilience also emerges as a critical factor in helping students cope with stressors.

Resilience is the ability to maintain one’s purpose and adapt positively despite enduring adversities and stressful events [22]. As a key protective factor, resilience mitigates negative psychological outcomes and mediates the relationship between stressful events and psychological well-being [23]. Studies found that resilience can buffer the negative effects of emotional abuse and depressive symptoms, with stronger effect in girls [24]. Resilient students are more likely to employ effective coping strategies, maintain positivity, and seek social support [25]. Besides, resilience could partially mediate the negative effect of bullying victimization on mental health [26] and higher resilience linked to fewer symptoms of depression and anxiety in bullied youth [27].

**Fig. 1:**
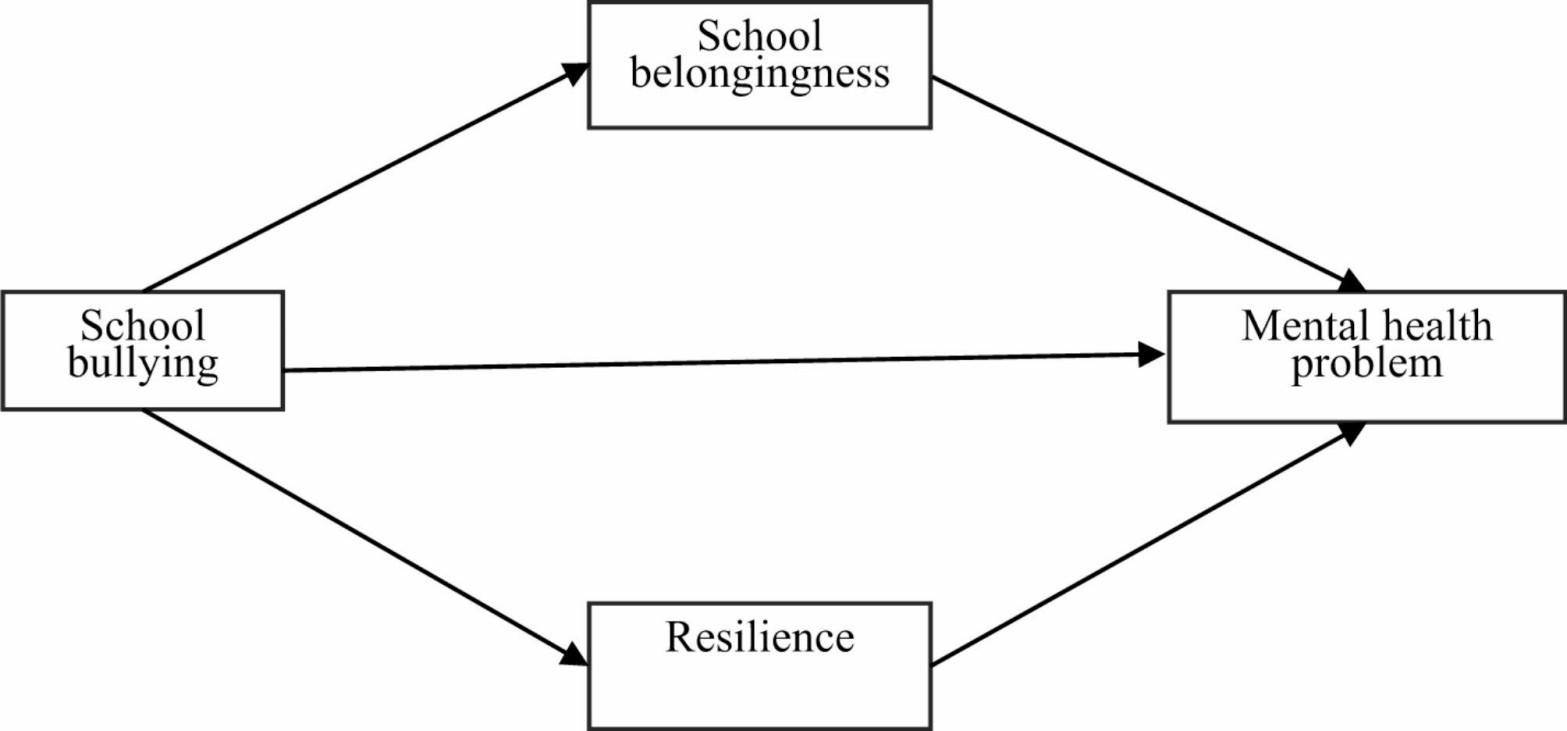
A hypothesized mediation model for bullying and mental health

Extensive researches have examined the impact of boarding schools on student development and the influence of bullying on mental health. However, few studies have specifically focused on how bullying affects boarding students or explored the potential mediating factors involved. This study aims to investigate the mental health impact of bullying on boarding secondary school students in Hebei Province, China, with a particular emphasis on the mediating roles of school belongingness and resilience in the bullying-mental health relationship (Fig. 1). By focusing on this vulnerable population, this research seeks to fill a gap in the literature by exploring how school belongingness and resilience function as mediators between bullying and negative mental health outcomes, offering valuable insights for targeted intervention strategies. The findings may also inform policies and best practices for creating safer and more supportive boarding school environments, and provide specific, actionable targets for enhancing student well-being.

## Methods

### Study design and sampling

The cross-sectional study was conducted June 2024 among boarding secondary school students in Hebei province, China. Stratified random cluster sampling was employed to administer a questionnaire survey across four boarding schools (three middle schools and one high school). Three classes were randomly selected from each grade in the middle schools and five from each grade in the high school, yielding 27 middle school and 15 high school classes. Due to school management policies and logistical considerations, electronic questionnaires were used for middle school students while paper questionnaires were administered to high school students. Participation was voluntary, and students completed the questionnaire with assistance from teachers or parents as needed. Epidata 3.1 software was used for double data entry and verification. Ethically approval was obtained from the Ethics Committee of Chines Academy of Medical Sciences (CAMS&PUMC-IEC-2024-039) on June 10th, 2024. The inclusion criteria included: (1) under 18 years old, (2) enrolled in a board secondary school, and (3) able to complete assessments independently with assistance. Exclusion criteria included significant cognitive impairment, life-threatening medical conditions or inability to cooperate with the study. Of the 1700 questionnaires collected, 1,560 were deemed valid (91.8% validity) after excluding those with obvious logical inconsistencies, missing key demographic information, or outliers.

### Measurements

The instruments used included the Delaware Bullying Victimization Scale, the Chinese Middle School Students Mental Health Scale (MSSMHS), the Chinese version of the Psychological Sense of School Membership (PSSM) Scale, and the 10-item Conner Davidson Resilience Scale (CD-RISC-10).

#### Demographic characteristics

Demographic characteristics included gender, age, grade, school, residence place, academic performance, family economic status, parents’ education level, and physical health status, etc.

#### Delaware bullying victimization scale

This study utilized the revised Chinese version of the Delaware Bullying Victimization Scale (Student Volume, 2016), consisting of 17 items across four dimensions: verbal, physical, social, and cyber bullying [28]. It is a six-point Likert scale (0 = never, 1 = occasionally, 2 = once or twice a month, 3 = once a week, 4 = many times a week, 5 = every day), with item 13 excluded from scoring. Higher scores indicate more severe bullying, with a score of ≥ 3 on any subscale considered indicative of bullying in that dimension. This study focused on verbal, physical and social bullying with a total score range of 0–60. Cronbach’s α = 0.941.

#### Middle School Students Mental Health Scale (MSSMHS)

The Chinese Middle School Students Mental Health Scale (MSSMHS) was specially developed to assess the psychological status and traits of Chinese middle school students, demonstrating strong reliability and validity [29]. The scale includes 10 subscales, of which 5 were selected based on research needs, including interpersonal sensitivity, depression, anxiety, learning pressure, and psychological imbalance. Each subscale contains 6 items, yielding a total of selected 30 items, rated on a 5-point Likert scale (1 = never, 5 = severe). The total score is the average of all item scores, with higher scores indicating greater mental health problems. A total score ≥ 2 is considered indicative of abnormal mental health (2 ~ 2.99 = mild, 3 ~ 3.99 = moderate, 4 ~ 4.99 = severe, 5 = very severe). Cronbach’s α = 0.967.

### Psychological Sense of School Membership (PSSM)

The Chinese version of the Psychological Sense of School Membership (PSSM-CR) [30] was used to assess adolescent students’ perceived sense of belonging or psychological membership in the school environment. It consists of 18 items on a six-point Likert scale ranging from 1 to 6 in descending order from “completely disagree” to “completely agree”. The total score ranges from 18 to 108, with higher scores indicating a stronger sense of school belonging. Cronbach’s α = 0.762.

#### 10 item Conner Davidson Resilience Scale (CD-RISC-10)

The 10 item Conner Davidson Resilience Scale (CD-RISC-10) [31] was used to assess participants’ psychological resilience. This scale is widely used due to its simplicity and clarity, and it has demonstrated good reliability and validity. Each item is scored on a 5-point scale (0 = never, 4 = always), with total scores ranging from 0 to 40, where higher scores indicating greater psychological resilience. Cronbach’s α = 0.956.

#### Statistical analysis

The descriptive analysis was used to summarize the collected data. Continuous variables were reported as means ± standard deviations (SD), and the categorical variables were reported as frequencies or percentages. Chi-square tests, t-tests, and ANOVA were employed to examine differences in demographic and related factors. Pearson correlation analysis explored relationships among bullying, mental health, school belongingness, and resilience. Path analysis was performed to examine the hypothesized mediation model with 5000 bootstrap sampling. The significance level was set at α = 0.05. All statistical analyses were performed using SPSS 26.0 and Amos 28.0.

## Results

### Sociodemographic characteristics of participants

The study sample include 755 boys (48.4%) and 805 girls (51.6%), with an average age of 15.9 ± 1.7 years. There were 245 students in grade 7 (15.7%), 256 in grade 8 (16.4%), 350 in grade 9 (22.4%), 226 in grade 10 (14.5%), 237 in grade 11 (15.2%), and 246 in grade 12 (15.8%). Additionally, 9.1% were only children, 67.1% attended public schools, 55.1% had moderate academic performance, 19.9% were class cadres, 62.4% were in good health, and 77.9% reported moderate family financial situation (Table 1).

### Results of school bullying and mental health status among participants

Abnormal mental health was reported by 527 students (33.8%), with 516 (33.1%) showing interpersonal sensitivity, 538 (34.5%) exhibiting depression symptoms, 611 (39.2%) experiencing anxiety symptoms, 765 (49.0%) reporting learning pressure, and 332 (21.3%) displaying psychological imbalance. The mean scores were 5.8 ± 9.0 for school bullying (higher among males, *P* = 0.025), 1.8 ± 0.8 for mental health problems (higher among females, *P* < 0.001), 76.3 ± 15.9 for school belongingness, and 24.1 ± 9.6 for psychological resilience (higher among males, *P* < 0.001). Verbal, physical, and social bullying were reported by 26.9%, 10.1%, and 15.4% of students, respectively, with a higher prevalence among males (*P* < 0.05) (Table 2).

Abnormal mental health status was reported by 61.9% of students who experienced verbal bullying, 73.9% of those who experienced physical bullying, and 70.5% of those who experienced social bullying. Students subjected to bullying demonstrated significantly higher prevalence of mental health problems (*P* < 0.001) (Table 3).

**Table 1:** Sociodemographic characteristics of participants

Variable	Male (*n* = 755)	Female (*n* = 805)	Total (*n* = 1560)
Age	15.8 ± 1.7	15.9 ± 1.8	15.9 ± 1.7
BMI	20.8 ± 3.6	20.2 ± 2.9	20.5 ± 3.3
BMI Group			
Wasting	92(12.2)	70(8.7)	162(10.4)
Normal weight	491(65.0)	626(77.8)	1117(71.6)
Overweight	122(16.2)	84(10.4)	206(13.2)
Obesity	50(6.6)	25(3.1)	75(4.8)
Grade			
Grade 7	134(17.7)	111(13.8)	245(15.7)
Grade 8	113(15.0)	143(17.8)	256(16.4)
Grade 9	177(23.4)	173(21.5)	350(22.4)
Grade 10	128(17.0)	98(12.2)	226(14.5)
Grade 11	100(13.2)	137(17.0)	237(15.2)
Grade 12	103(13.6)	143(17.8)	246(15.8)
Only-Child Status	91(12.1)	51(6.3)	142(9.1)
Household Registration Status			
Urban	138(18.3)	136(16.9)	274(17.6)
Rural	617(81.7)	669(83.1)	1286(82.4)
Type of School Attended			
Public	480(63.6)	567(70.4)	1047(67.1)
Private	275(36.4)	238(29.6)	513(32.9)
Academic Performance			
Top ten	182(24.1)	197(24.5)	379(24.3)
Average	411(54.4)	448(55.7)	859(55.1)
Below average	162(21.5)	160(19.9)	322(20.6)
Class Cadres	156(20.7)	154(19.1)	310(19.9)
Physical health status			
Healthy	502(66.5)	471(58.5)	973(62.4)
Occasionally ill	224(29.7)	269(33.4)	493(31.6)
Frequently ill	29(3.8)	65(8.1)	94(6.0)
Family Financial Situation			
Affluent	18(2.4)	34(4.2)	52(3.3)
Moderate	578(76.6)	638(79.3)	1216(77.9)
Low-income	120(15.9)	116(14.4)	236(15.1)
Impoverished	39(5.2)	17(2.1)	56(3.6)
Father’s Education Level			
Primary school	177 (23.4)	158 (19.6)	335 (21.5)
Junior high school	402 (53.2)	495 (61.5)	897 (57.5)
High school	105 (13.9)	98 (12.2)	203 (13.0)
Junior college and above	71 (9.4)	54 (6.7)	125 (8.0)
Mother’s Education Level			
Primary school	221 (29.3)	209 (26.0)	430 (27.6)
Junior high school	400 (53.0)	461 (57.3)	861 (55.2)
High school	77 (10.2)	87 (10.8)	164 (10.5)
Junior college and above	57 (7.5)	48 (6.0)	105 (6.8)

**Table 2:** Results of school bullying and mental health status among participants

	Male (*n* = 755)	Female (*n* = 805)	Total (*n* = 1560)	*P*
School bullying	6.3 ± 9.9	5.3 ± 8.2	5.8 ± 9.0	0.025
Verbal bullying subscale	3.2 ± 4.3	2.6 ± 3.4	2.9 ± 3.9	0.003
Verbal bullying	225 (29.8)	195 (24.2)	420 (26.9)	0.013
Physical bullying subscale	1.6 ± 3.2	1.0 ± 2.5	1.3 ± 2.8	< 0.001
Physical bullying	104 (13.8)	53 (6.6)	157 (10.1)	< 0.001
Social bullying subscale	1.6 ± 3.2	1.8 ± 3.0	1.7 ± 3.1	0.280
Social bullying	102 (13.5)	139 (17.3)	241 (15.4)	0.040
Mental Health Problems	1.7 ± 0.8	1.9 ± 0.8	1.8 ± 0.8	< 0.001
Interpersonal sensitivity	1.7 ± 0.8	1.8 ± 0.8	1.8 ± 0.8	0.001
Depression	1.7 ± 0.8	1.9 ± 0.9	1.8 ± 0.9	< 0.001
Anxiety	1.8 ± 0.9	2.0 ± 1.0	1.9 ± 1.0	< 0.001
Feeling of learning pressure	2.0 ± 1.0	2.2 ± 1.0	2.1 ± 1.0	< 0.001
Psychological imbalance	1.5 ± 0.7	1.5 ± 0.7	1.5 ± 0.7	0.865
Mental health status				0.002
Normal	537 (71.1)	496 (61.6)	1033 (66.2)	
Mild abnormal	149 (19.7)	215 (26.7)	364 (23.3)	
Moderate abnormal	56 (7.4)	80 (9.9)	136 (8.7)	
Severe abnormal	9 (1.2)	12 (1.5)	21 (1.3)	
More severe abnormal	4 (0.5)	2 (0.2)	6 (0.4)	
Interpersonal sensitivity				0.013
normal	534 (70.7)	510 (63.4)	1044 (66.9)	
Mild abnormal	144 (19.1)	193 (24.0)	337 (21.6)	
Moderate abnormal	62 (8.2)	88 (10.9)	150 (9.6)	
Severe abnormal	8 (1.1)	11 (1.4)	19 (1.2)	
More severe abnormal	7 (0.9)	3 (0.4)	10 (0.6)	
Depression symptom				< 0.001
normal	543 (71.9)	479 (59.5)	1022 (65.5)	
Mild abnormal	142 (18.8)	197 (24.5)	339 (21.7)	
Moderate abnormal	47 (6.2)	101 (12.5)	148 (9.5)	
Severe abnormal	15 (2.0)	22 (2.7)	37 (2.4)	
More severe abnormal	8 (1.1)	6 (0.7)	14 (0.9)	
Anxiety symptom				< 0.001
normal	499 (66.1)	450 (55.9)	949(60.8)	
Mild abnormal	151 (20.0)	191 (23.7)	342(21.9)	
Moderate abnormal	72 (9.5)	106 (13.2)	178(11.4)	
Severe abnormal	21 (2.8)	48 (6.0)	69(4.4)	
More severe abnormal	12 (1.6)	10 (1.2)	22(1.4)	
Learning pressure				< 0.001
normal	434 (57.5)	361 (44.8)	795 (51.0)	
Mild abnormal	186 (24.6)	252 (31.3)	438 (28.1)	
Moderate abnormal	95 (12.6)	128 (15.9)	223 (14.3)	
Severe abnormal	27 (3.6)	48 (6.0)	75 (4.8)	
More severe abnormal	13 (1.7)	16 (2.0)	29 (1.9)	
Psychological imbalance				0.152
normal	597 (79.1)	631 (78.4)	1228 (78.7)	
Mild abnormal	104 (13.8)	136 (16.9)	240 (15.4)	
Moderate abnormal	42 (5.6)	30 (3.7)	72 (4.6)	
Severe abnormal	6 (0.8)	5 (0.6)	11 (0.7)	
More severe abnormal	6 (0.8)	3 (0.4)	9 (0.6)	
School belongingness	77.1 ± 16.4	75.6 ± 15.5	76.3 ± 15.9	0.052
Resilience	25.0 ± 9.9	23.2 ± 9.2	24.1 ± 9.6	< 0.001

**Table 3:** Prevalence of each mental health problem in three bullying types

	Verbal bullying		Physical bullying		Social bullying	
	Yes (*n* = 420)	No (*n* = 1140)	Yes (*n* = 157)	No (*n* = 1403)	Yes (*n* = 241)	No (*n* = 1319)
Mental health status						
Normal	160 (38.1)	873 (76.6)	41 (26.1)	992 (70.7)	71 (29.5)	962 (72.9)
Mild abnormal	157 (37.4)	207 (18.2)	58 (36.9)	306 (21.8)	88 (36.5)	276 (20.9)
Moderate abnormal	81 (19.3)	55 (4.8)	40 (25.5)	96 (6.8)	59 (24.5)	77 (5.8)
Severe abnormal	16 (3.8)	5 (0.4)	12 (7.6)	9 (0.6)	17 (7.1)	4 (0.3)
More severe abnormal	6 (1.4)	0 (0.0)	6 (3.8)	0 (0.0)	6 (2.5)	0 (0.0)
Interpersonal sensitivity						
Normal	160 (38.1)	884 (77.5)	40 (25.5)	1004 (71.6)	74 (30.7)	970 (73.5)
Mild abnormal	144 (34.3)	193 (16.9)	54 (34.4)	283 (20.2)	74 (30.7)	263 (19.9)
Moderate abnormal	94 (22.4)	56 (4.9)	46 (29.3)	104 (7.4)	71 (29.5)	79 (6.0)
Severe abnormal	14 (3.3)	5 (0.4)	9 (5.7)	10 (0.7)	14 (5.8)	5 (0.4)
More severe abnormal	8 (1.9)	2 (0.2)	8 (5.1)	2 (0.1)	8 (3.3)	2 (0.2)
Depression symptom						
Normal	176 (41.9)	846 (74.2)	48 (30.6)	974 (69.4)	80 (33.2)	942 (71.4)
Mild abnormal	128 (30.5)	211 (18.5)	48 (30.6)	291 (20.7)	72 (29.9)	267 (20.2)
Moderate abnormal	84 (20.0)	64 (5.6)	38 (24.2)	110 (7.8)	62 (25.7)	86 (6.5)
Severe abnormal	20 (4.8)	17 (1.5)	12 (7.6)	25 (1.8)	15 (6.2)	22 (1.7)
More severe abnormal	12 (2.9)	2 (0.2)	11 (7.0)	2 (0.2)	12 (5.0)	2 (0.2)
Anxiety symptom						
normal	148 (35.2)	801 (70.3)	42 (26.8)	907 (64.6)	69 (28.6)	880 (66.7)
Mild abnormal	131 (31.2)	211 (18.5)	49 (31.2)	293 (20.9)	65 (27.0)	277 (21.0)
Moderate abnormal	87 (20.7)	91 (8.0)	36 (22.9)	142 (10.1)	62 (25.7)	116 (8.8)
Severe abnormal	37 (8.8)	32 (2.8)	14 (8.9)	55 (3.9)	29 (12.0)	40 (3.0)
More severe abnormal	17 (4.0)	5 (0.4)	16 (10.2)	6 (0.4)	16 (6.6)	6 (0.5)
Learning pressure						
Normal	118 (28.1)	677 (59.4)	33 (21.0)	762 (54.3)	56 (23.2)	739 (56.0)
Mild abnormal	150 (35.7)	288 (25.3)	47 (29.9)	391 (27.9)	77 (32.0)	361 (27.4)
Moderate abnormal	95 (22.6)	128 (11.2)	44 (28.0)	179 (12.8)	63 (26.1)	160 (12.1)
Severe abnormal	36 (8.6)	39 (3.4)	17 (10.8)	58 (4.1)	25 (10.4)	50 (3.8)
More severe abnormal	21 (5.0)	8 (0.7)	16 (10.2)	13 (0.9)	20 (8.3)	9 (0.7)
**Psychological imbalance**						
Normal	232 (55.2)	996 (87.4)	59 (37.6)	1169 (83.3)	116 (48.1)	1112 (84.3)
Mild abnormal	122 (29.0)	118 (10.4)	53 (33.8)	187 (13.3)	69 (28.6)	171 (13.0)
Moderate abnormal	49 (11.7)	23(2.0)	30 (19.1)	42 (3.0)	40 (16.6)	32 (2.4)
Severe abnormal	9 (2.1)	2 (0.2)	7 (4.5)	4 (0.3)	8 (3.3)	3 (0.2)
More severe abnormal	8 (1.9)	1 (0.1)	8 (5.1)	1 (0.1)	8 (3.3)	1 (0.1)

### The mediation effect of school belongingness and resilience

Correlation analysis showed that school belongingness was positively correlated with psychological resilience (*r* = 0.424) and negatively correlated with school bullying and mental health problems (*r*=-0.449, *r*=-0.619). Psychological resilience was negatively correlated with school bullying and mental health problems (*r*=-0.175, *r*=-0.338). School bullying was positively associated with mental health problems (*r* = 0.539) (Table 4).

**Fig. 2:**
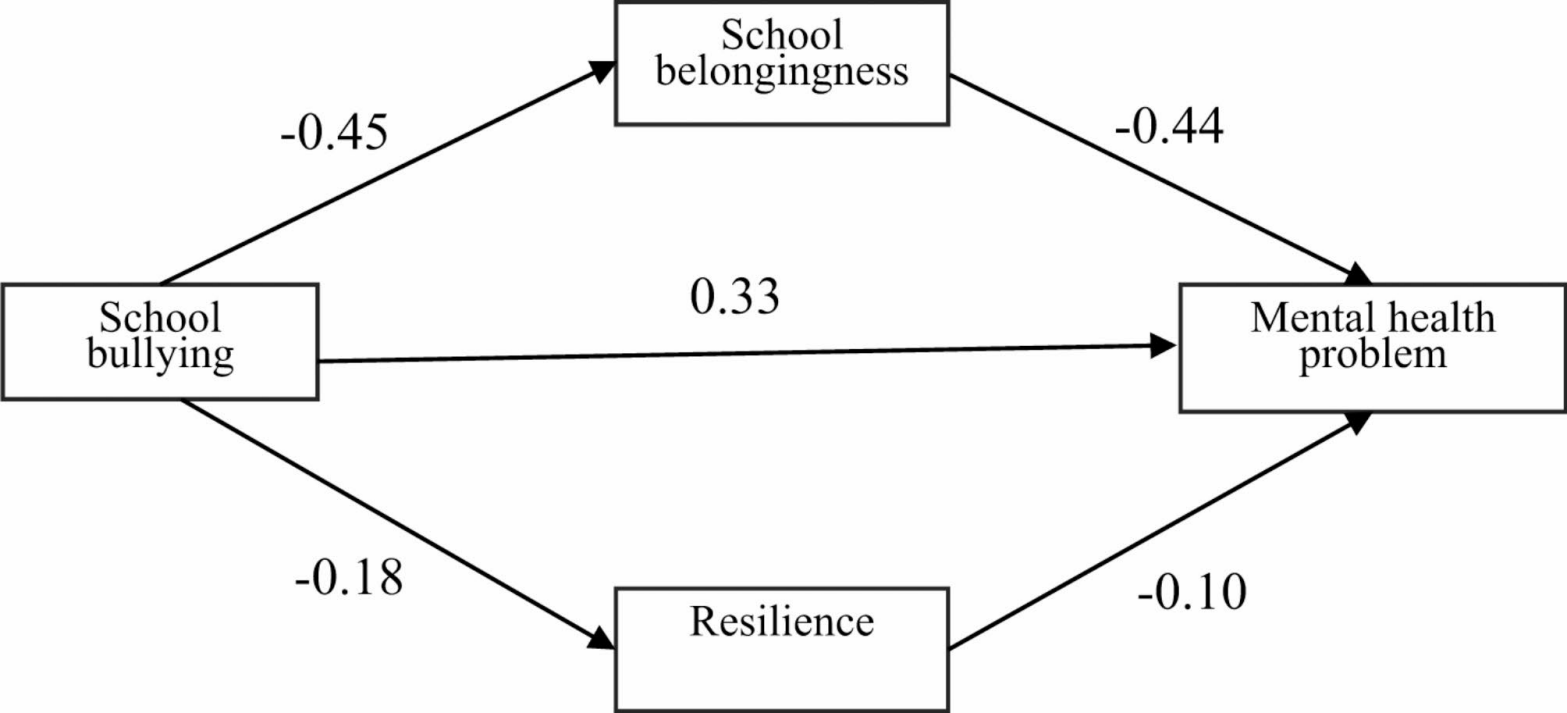
The Mediating Role of School Belongingness and Resilience

Path analysis was conducted to test the hypothesized mediation model, with mental health problems as the dependent variable, school bullying as the independent variable, and school belongingness and resilience as mediators (Fig. 2). The results showed that school bullying positively predicted mental health problems significantly (β = 0.334, *P* < 0.001), while school belongingness and psychological resilience negatively predicted mental health problems significantly (β=-0.435, -0.100; *P* < 0.001) (Table 5). Mediation analysis indicated that school belongingness and resilience partially mediated the relationship between bullying and mental health, accounting for 35.7% and 3.2% of the total effect, with mediating effect values of 0.017 (0.014–0.020) and 0.002 (0.001–0.003), respectively (Table 6).

**Table 4:** Correlational analysis of school belongingness, psychological resilience, school bullying and mental health problems (r)

	School belongingness	Resilience	School bullying	Mental health problem
School belongingness	1			
Resilience	0.424**	1		
School bullying	-0.449**	-0.175**	1	
Mental health problem	-0.619**	-0.338**	0.539**	1

Note: **P < 0.01

**Table 5:** Direct effects test of school belongingness, psychological resilience, school bullying, and mental health problems

Pathways	Unstandardized estimates	Standardized estimates	S.E.	C.*R*.	*P*
School bullying → School belongingness	-0.790	-0.449	0.040	-19.836	< 0.001
School bullying →Resilience	-0.185	-0.175	0.026	-7.025	< 0.001
School bullying→ Mental health problem	0.029	0.334	0.002	15.871	< 0.001
School belongingness →Mental health problem	-0.022	-0.435	0.001	-20.922	< 0.001
Resilience →Mental health problem	-0.008	-0.100	0.002	-5.302	< 0.001

**Table 6:** Mediating effect analysis of school belongingness and psychological resilience on school bullying and mental health problem

Pathways		Estimates	95%CI		*P*
			Lower	Upper	
Direct effect	School bullying → Mental health problem	0.029	0.023	0.035	≤ 0.001
Mediating effect	School bullying → School belongingness → Mental health problem	0.017	0.014	0.020	≤ 0.001
	School bullying → Resilience → Mental health problem	0.002	0.001	0.004	≤ 0.001
Total effect		0.048	0.042	0.053	≤ 0.001

## Discussion

This study examined the prevalence of school bullying among boarding students in Hebei Province, China, and its impact on mental health, focusing on the mediating roles of school belongingness and psychological resilience. The findings highlight the psychosocial challenges faced by adolescents in boarding schools and underscore the importance of targeted interventions to mitigate the effects of bullying. Besides, the findings further indicate that school belongingness and psychological resilience act as important mediators in the relationship between bullying and mental health issues, helping to explain the psychological consequences of bullying.

### Prevalence of bullying and mental health impact of boarding students

The study found that a substantial proportion of boarding students experienced bullying in Hebei Province, China, with 26.9% reporting verbal bullying, 10.1% physical bullying, and 15.4% relational bullying. These rates align with global estimates, underscoring the persistence of bullying across diverse educational contexts. What’s more, gender differences were notable, with boys reporting higher rates of verbal and physical bullying than girls (*P* < 0.05). This aligns with existing research suggesting that boys were more likely to experience physical forms of bullying, whereas girls were more susceptible to relational aggression [32]. Tailored intervention strategies should address these gender-specific patterns.

This study demonstrates a significant association between various types of bullying and mental health issues among boarding students, underscoring the pervasive impact of bullying on psychological well-being. Students experiencing physical bullying reported significantly higher levels of severe anxiety and depressive symptoms, consistent with prior research indicating that frequent bullying increases the risk of long-term psychological problems, including depression, anxiety and psychosomatic symptom [33]. Longitudinal research further revealed that individuals bullied in childhood face a significantly higher risk of developing psychiatric disorders in adulthood, such as anxiety, depression, and suicidality [34]. Students who experience verbal bullying exhibited severe psychological imbalances, suggesting a distinct effect on self-perception and emotional regulation. Moreover, bullying was shown to disrupt interpersonal relationships and psychological stability, contributing to increased relational problems and psychological imbalance among those who have been bullied. The higher academic stress reported by bullied students underscores the broader impact of bullying on overall well-being. The positive correlation between bullying and psychological distress suggests that bullying acts as a chronic stressor, potentially triggering or exacerbating mental health problems, consistent with the stress-process model [35]. The adverse mental health outcomes identified highlight the importance of comprehensive anti-bullying programs in schools that not only aim to prevent bullying but also provide robust support systems for victims.

### Mediating roles of school belongness and psychological resilience

School belongingness was identified as a significant mediator in the relationship between bullying and mental health problems, with 35.7% of the total effect of bullying on mental health being mediated by students’ sense of belongingness to their school. This underscores its critical role in promoting adolescent well-being. Consistent with prior research, a strong sense of school belongingness was associated with lower levels of depression and anxiety among, even in the presence of bullying [36]. Studies suggest that school belongingness serves as both a protective and promotive factor, enabling students to cope more effectively with adversities such as bullying, thereby reducing psychological distress [37, 38]. As a critical socio-ecological factor, school belongingness fosters resilience by encouraging positive coping strategies, which help adolescents overcome challenges, such as bullying, while improving their overall well-being [39–41]. The negative correlation between school belongingness and both bullying and mental health issues in this study further supports the idea that fostering a sense of belongingness may mediate the harmful effects of bullying. Students who feel more connected to their school community are likely to experience higher levels of social support, which can help them cope with negative impacts of peer victimization [42]. Given the strong mediating role of school belongingness, schools should prioritize fostering an inclusive environment that promotes positive school climates, strengthens student-teacher relationships, and encourages peer support.

In addition to school belongingness, resilience also emerged as a significant mediator in the relationship between bullying and mental health, although its mediating effect was relatively modest compared to school belongingness, accounting for 3.2% of the total effect. This aligns with resilience theory, which emphasizing that resilient individuals are better equipped to manage stress and recover from negative experiences, including bullying [43, 44]. However, our findings contrast with a study in Yunnan Province, China, where resilience was found to account for a larger proportion of the relationship between bullying and anxiety [45]. The difference suggests that resilience’s protective role may vary across different contexts and populations, as it is not merely an individual trait but a complex, dynamic process influenced by individual, relational, and contextual factors [46, 47]. The stress-coping model further support this view, emphasizing that resilience is part of a broader coping strategy, where internal resources (such as resilience) interact with external factors (such as school support systems) to buffer against stressors like bullying [48]. Therefore, the modest mediating effect of resilience in our study may reflect the fact that its protective effects are most pronounced when combined with other factors, such as school belongingness, which creates a supportive environment for emotional and psychological resilience. Given resilience’s context-dependent nature, we suggest that interventions should not only focus on enhancing individual resilience but also address the broader relational and environmental factors that contribute to it [49]. Therefore, schools could adopt a multi-tiered approach, promote a positive school climate while offering targeted support for students who are at higher risk of bullying and mental health problems. Additionally, establishing a national platform integrating real-time monitoring, preventive education, and intervention functions is recommended to effectively address bullying and mental health issues, ensuring a coordinated, nationwide response that enhance both the effectiveness and reach of anti-bullying initiatives.

There are several limitations of this study. First, the cross-sectional design restricts the ability to establish causal relationships between the variables examined, suggesting that future research should employ longitudinal designs to better understand the temporal relationships between bullying, school belongingness, resilience, and mental health outcomes. Second, while this study focused on the mediating roles of school belongingness and resilience, future research should explore other potential mediators and better understand the complex mechanisms that explain the relationship between bullying and mental health outcomes. Third, the study’s reliance on a sample drawn from Hebei Province, China, which limits the generalizability of the findings. Future studies should aim to replicate this research in diverse settings to assess their boarding applicability. Fourth, although all scales demonstrated high internal consistency, this study used path analysis with observed variables, which does not account for measurement error at the item level. This choice was made to avoid increasing model complexity, as the original scales contained many items, and to prioritize model simplicity for better interpretability and computational efficiency. While the high reliability of the scales reduces concerns about measurement error, future research could benefit from structural equation modeling with latent constructs to enhance measurement precision. Finally, the study’s exclusive reliance on self-reported measures may introduce biases such as social desirability and recall bias. To obtain a more comprehensive understanding of bullying and its impact on mental health, future research should incorporate additional data sources, such as teacher reports, peer assessments, and behavioral observations.

## Conclusions

In conclusion, this study reveals the significant impact of school bullying on mental health of boarding secondary school students in Hebei Province, China. It highlights the critical roles of school belongingness and psychological resilience as mediators that explain the effects of bullying on mental health. These insights have important implications for educational policy and practice, indicating that schools should implement comprehensive anti-bullying programs that not only address bullying behaviors but also promote positive school climates and supportive relationships among students and staff. By prioritizing interventions that enhance students’ sense of belonging and connection to their school community, alongside resilience training, schools can better mitigate the negative effects of bullying and support students’ overall well-being.

## Data Availability

No datasets were generated or analysed during the current study.

## Abbreviations

MSSMHS: The Chinese Middle School Students Mental Health Scale
PSSM-CR: The Chinese version of the Psychological Sense of School Membership Scale
CD-RISC-10: 10 item Conner Davidson resilience scale
SPSS: Statistical package for the social sciences
ANOVA: Analysis of variance
UNESCO: United Nations Educational, Scientific and Cultural Organization
SD: Standard deviation
BMI: Body mass index
S.E.: Standard error
C.R.: Critical ratio
CI: Confidence interval
SEL: Social-emotional learning
